# Type 2 diabetes severity in the workforce: An occupational sector analysis using German claims data

**DOI:** 10.1371/journal.pone.0309725

**Published:** 2024-09-27

**Authors:** Batoul Safieddine, Julia Grasshoff, Stefanie Sperlich, Jelena Epping, Siegfried Geyer, Johannes Beller

**Affiliations:** Medical Sociology Unit, Hannover Medical School, Hannover, Germany; Johns Hopkins University Bloomberg School of Public Health, UNITED STATES OF AMERICA

## Abstract

**Background:**

Individuals of working age spend a significant amount of time at the workplace making it an important context for disease prevention and management. The temporal development and prevalence of T2D have been shown to differ in the working population based on gender, age group and occupational sector regardless of socioeconomic status. Given potential differences in risk factors associated with different work environments, this study aims to define vulnerable occupational groups by examining T2D severity and its trends in working men and women with T2D of two age groups and among nine occupational sectors.

**Methods:**

The study is based on claims data of the statutory health insurance provider AOKN. The study population consisted of all insured working individuals with T2D. T2D severity was measured using the adapted diabetes complications severity index—complication count (DCSI-CC). Mean DCSI-CC scores were calculated over four time periods between 2012 and 2019 for men and women of the age groups 18–45 and 46+ years and among nine occupational sectors. Trends of DCSI-CC were investigated using ordinal logistic regression analyses to examine the effect of time-period on the odds of having higher DCSI scores.

**Results:**

Overall, there was a significant rise in T2D severity over time in working men and women of the older age group. Moreover, the study displayed occupational sector differences in T2D severity and its trends. Over all, working men of all sectors had higher DCSI-CC scores compared to working women. Individuals working in the sector “Transport, logistics, protection and security” and “Construction, architecture, measuring and building technology” had higher T2D severity, while those working in the “Health sector, social work, teaching & education” had relatively lower T2D severity. There was a gender-specific significant increase over time in T2D severity in the above-mentioned occupational sectors.

**Conclusion:**

The study displayed gender, age group and occupational sector differences in T2D severity and its trends. Working individuals could thus benefit from personalized prevention interventions that consider occupational contexts. As a next step, examining T2D trends and severity in specific occupations within the vulnerable occupational sectors is needed.

## Introduction

Type 2 diabetes (T2D) is a growing global pandemic and belongs to the most common chronic diseases worldwide [1, 2]. As life expectancy has been on the rise during the last decades, the prevalence of manageable chronic diseases like T2D has been drastically increasing over time [3]. This development could have a substantial impact on employment and the labour force, as evidence points towards an increase in the prevalence of T2D also in individuals of working age [4]. However, the extent to which T2D could be affecting the wellbeing of working individuals in terms of complications and severity needs to be examined to infer adequate conclusions.

In studies from social epidemiology, occupation is often used as an indicator of socio-economic position, social prestige or as a proxy for different degrees of job autonomy [5, 6]. The substantive content of work often remains unconsidered, although different occupations may be associated with different risk profiles for the onset of T2D. These profiles may also change over time due to technological and social developments. Individuals working in occupations that require physical activity might be naturally exposed to a protective environment despite the level of health literacy as an important determinant of T2D and its complications [7, 8]. On the other hand, individuals with high educational level and health literacy might be working in white-collar positions associated with high levels of distress [9] and little opportunity for adequate physical activity [10, 11]. Within this context, digitalization and the use of information and communication technologies also play an important role in health trends of working individuals during the last few decades, as many jobs are requiring less physical activity and activity levels may decrease over time [12]. Moreover, evidence points towards an overall increase in sedentary behaviour such as prolonged sitting time [13] and insufficient physical activity [14] in individuals of working age. Thus, it is of major importance to investigate health trends in different occupations despite their hierarchy and social class levels.

A recent study investigated the temporal development of T2D in the employed population and among different occupational sectors. The study was based on German statutory health insurance data, and reported differential trends among different age groups of employed individuals. While the prevalence of T2D seemed to be decreasing over an eight-year period between 2012 and 2019 in older employed individuals (46+ years), the prevalence of T2D was significantly increasing among younger working men and women (18–45 years). Stratifying the working population by occupational sector, and regardless of the socioeconomic status, the study revealed that certain occupational sectors have higher risks for T2D compared to others. For example, individuals working in the occupational sector “Agriculture” had by far the lowest T2D rates, while women working in “Health sector, social work, teaching & education” and men working in the “Transport, logistics, protection and security” sector had highest T2D rates [15]. Nevertheless, severity of T2D can vary widely as the development of T2D complications is dependent on the lifestyle and medicinal management. Health literacy, physical activity associated with daily routines as well as stress are related factors that might differ widely among occupational sectors. This might indicate that individuals with T2D working in different occupational sectors might have different T2D severity levels, which could have important implications related to T2D prevention. For example, while women working in the health sector have relatively high rates of T2D compared to other sectors [15], they might be suffering from less T2D complications due to better health literacy.

Thus, investigating the severity of T2D among different occupational sectors is essential to identify possible risk factors and gaps in prevention interventions. It could help in tailoring prevention interventions to address target groups accurately. Moreover, as differential trends in T2D have been observed in the employed population among different age groups [15], it is essential to understand whether trends in T2D severity would follow similar patterns. For example, while T2D rates are increasing in younger employed individuals, the severity of T2D in this group might be decreasing over time due to medical progress and improved prevention. In fact, research pointed towards an overall expansion of morbidity in individuals with T2D due to having higher rates of most comorbidities over time [16, 17]. However, it remains open whether this applies when stratifying the employed population by age group, and whether it equally applies to individuals working in different occupational contexts.

Based on the results of the mentioned study [15], and using the same database, this study aims to expand the analyses by examining the trends of T2D severity in the employed population. Moreover, the study aims to examine how T2D severity and its trends differ among nine occupational sectors. This would provide the literature with evidence on occupational sectors that need to be focused upon in depth in further studies, as well as information needed so that prevention interventions could be designed to tailor vulnerable groups. To the best of our knowledge, this is the first study to consider T2D severity among different occupational sectors, and the first to consider the time aspect as well as detailed stratification by gender and age to help capture vulnerable groups. Specifically, the study aims to examine:

1. T2D severity in working men and women of two age groups and in nine occupational sectors
2. Trends of T2D severity between 2012 and 2019 in working men and women of two age groups and in nine occupational sectors

## Methods

### Database

The study is based on data from the German statutory health insurance provider: the “Allgemeine Ortskrankenkasse Niedersachsen” (AOKN). The data were anonymized by AOKN before we had any access to them. Federal law regulates the use of this sort of data for scientific purposes. The data protection officer of the Statutory Local Health Insurance of Lower Saxony has approved its use at our institute within the frame of the ongoing research project. The data this study is based on were available to us since 2014 for the year 2012, with annual updates including the data for each subsequent year.

AOKN is the largest in the state of Lower Saxony, insuring around 3 million individuals, around a third of the inhabitants in this state. It includes all in- and outpatient diagnoses, performed medical services and prescribed treatments. The diagnoses are coded according to the German 10^th^ version of the international classification of disease (ICD-10-GM).

### Definition of T2D

The study population included all AOKN insured working men and women with T2D between 2012 and 2019. Even though T2D is assigned the ICD-10-GM code “E11”, additional criteria were considered in order to limit potential errors associated with erroneous or double coding which can be present in some cases. In each time period, individuals were considered to have prevalent T2D if among other diabetes diagnoses, “E11” was coded most frequently. Since over 90% of diabetes cases are T2D, individuals were also considered to have prevalent T2D if “E14”, which corresponds to the diabetes type “undefined”, was most frequently coded. Moreover, individuals were also considered to have T2D if “E10”, which corresponds to type 1 diabetes, was most frequently coded, but no insulin prescriptions were coded in the corresponding time period. As a next step, in each time period, diagnoses were considered to be valid only of they were coded in at least two quarter of that period. An exception to this criterion was applied to individuals who were insured for only one quarter in the corresponding period.

### Occupational sector

In AOKN, occupation is coded according to the newest classification of occupations (KldB2010) as provided by the German Federal Employment Agency (Bundesagentur für Arbeit) [18]. This classification was applied for coding occupation from the year 2012. It considers additional aspects such as vocational training and education, and is closer to the international standard classification of occupations (ISCO) compared to earlier versions. KldB2010 classifies more than 1200 specific occupations that lie under ten occupational sectors. These occupational sectors are: “Military”, “Agriculture”, “Extraction of raw material, production and manufacturing”, “Construction, architecture, measuring and building technology”, “Natural sciences, geography, information”, “Transport, logistics, protection and security”, “Commercial, trade, distribution and tourism”, “Corporate organization, accounting, law and administration”, “Health sector, social work, teaching & education” and “Humanities, culture and design”. Health care costs of employed men and women in the occupational sector “Military” are covered by federal aid (Beihilfe) and private insurance. Thus, the data included a very small number of insured individuals working in this sector, so this occupational sector was excluded from the analyses. The study stratified the analyses by the nine remaining occupational sectors. In some cases, individuals worked in several occupational sectors in one time period. In this case, the occupational sector of the longest duration per time period was considered.

### Time period

At the time of the study, the data was available for the years 2005 to 2019. Since only the new classification of occupations was considered (KldB2010) which was applied in 2012, the study used the data starting from the year 2012. Four time periods between 2012 and 2019 were considered as follows: 2012–2013 (p1), 2014–2015 (p2), 2016–2017 (p3) and 2018–2019 (p4). To improve comparability of time periods, T2D, complications, diabetes severity as well as affiliation to occupational sector were defined newly in each time period using the described criteria.

### Diabetes severity

Diabetes severity was measured by the diabetes complications severity index—complication category count (DCSI-CC). The original diabetes complications severity index (DCSI) is a scoring system developed by Young and colleagues in 2008 in order to quantify the overall severity of diabetes [19]. It considers the following seven categories of complications: cardiovascular disease, nephropathy, retinopathy, peripheral vascular disease, stroke, neuropathy, and metabolic complications. Using laboratory data, the DCSI score quantifies the severity of each complication by grading it with “1” for non severe and “2” for severe. DCSI scores have been validated against outcomes like risks of mortality and hospitalization, and have been shown to be a valid measure of diabetes severity adapted by researchers in numerous studies to quantify diabetes severity [20–23]. Nevertheless, since claims data does not include laboratory results, Chang and colleagues (2012) proposed and tested the use of the complication category count based on the DCSI definition of complications *using claims data*, but without considering the severity of each complication category. The validation analyses lead to the conclusion that DCSI-CC is a good measure of diabetes severity, and performs similar to the original DCSI score that considers laboratory data, as risk ratios for hospitalizations were similar using both measures [24]. Moreover, Chang et al (2012) also reported in another study that DCSI-CC is a better indicator than the simple complications count in predicting health care costs in T2D [25].

We used the DSCI-CC to compare the severity of T2D among different occupational sectors and over time. The DSCI-CC provides a score of “1” for each complication category if complications belonging to this category are present. Consequently, the DSCI-CC score can range between “0” and “7”. While the original DCSI was developed using the ninth edition of the international classification of diseases (ICD-9), Glasheen and colleagues [26] and Wicke and colleagues [27] translated the ICD-9 version of the DCSI to ICD-10. Our study used the ICD-10 translation of Wicke et al. who translated the ICD-9 DCSI to the German version of ICD-10 and showed its validity in predicting hospitalization, mortality and health care costs using claims data [27]. ICD-10 codes for the seven comorbidity categories are found in supplementary material (S1 Table).

Similar to the definition of diabetes, diagnoses of the complications were considered valid if they were coded in at least two quarters of the considered time period. Exceptions for this criterion applied if individuals were insured for only one quarter.

### Age groups

The study stratified the analyses by two age groups: 18–45 years and >45 years, based on various considerations. Firstly, the age of 45 is recognized as a pivotal point for the onset of T2D [28], as physiological changes during early to middle adulthood increase the risk of T2D [29, 30]. Additionally, different phases of adulthood present unique social and biological challenges affecting risk profiles and the effectiveness of interventions [31]. The way one’s occupation can impact health can also differ depending on the life stage. As early adulthood is defined as the age range between 18–35 years, it was methodologically not optimal to stratify the age accordingly, or to consider more than two age groups, because doing so would result in a small N in certain groups due to the detailed stratification applied in the study (age, gender and nine occupation groups). Thus, splitting the age groups by 45 years was a good compromise. In fact, splitting the age group at 45 years also aligns with the phase of life often associated with parenting, considering the average age for having a first child in Germany is between 30–33 years [32, 33]. This division enables the exploration of the distinct social and familial challenges faced by working adults at these stages, and how these challenges may differentially affect their lifestyle, well-being and health.

### Statistical analyses

In the first line of analysis, mean DCSI-CC scores and confidence intervals were calculated for all working men and women in the two age groups 18–45 years and >45 years in the two time periods p1 and p4. Then, ordinal logistic regression analysis was applied to examine the trends by examining the effect of time period (being in p4 compared to p1) on the odds of having at least one additional score point in DCSI-CC [34]. Separate models were calculated for men and women and each age group, creating four models for this part of analysis. In all models, DSCI score in its ordinal categories (0–7) was the dependant variable, and time period with the two categories p1 and p4 was the main independent variable, with p1 being the reference group. All models adjusted for age within each age group and insurance duration. Age was added as a continuous variable and corresponds to the age of individuals in the examined subgroups, since the age groups were relatively wide. Insurance duration of individuals within each subgroup and time period was added to the models in order to correct for potential censoring, as not all individuals are insured (and thus observed) for the same amount of time.

In a second step, Mean DCSI-CC scores and ranges were calculated for men and women in each occupational sector in the four time periods between 2012–2019. Then, ordinal logistic regression analyses was used to examine the effect of being in p4 compared to p1 on the odds of having at least one additional score point in DCSI-CC. A separate logistic regression analysis was done for each occupational sector, gender and age group, resulting in 36 models. All models adjusted for age and insurance duration.

In all ordinal logistic regression models applied in this study, cluster-robust standard errors were used to correct for possible autocorrelation [35] due to the possibility of having same individuals in more than one period.

All analyses in this study were conducted using the statistics software STATA version 16.0.

### Ethical approval and consent to participate

This study did not require ethical approval. The analyses were performed using a pre-existing claims dataset created as part of the routine administrative activities of a statutory health insurance provider. Its scientific use is regulated by German law in the German Social Code “Sozialgesetzbuch”. The data protection officer of the Local Statutory Health Insurance of Lower Saxony-AOK Niedersachsen (Germany) has given permission for this study to use the data for scientific purposes. The study was conducted in accordance with the Declaration of Helsinki. Informed consent was not needed since the database in this study is a pre-existing anonymized claims dataset and contact to patients did not exist in any form.

## Results

The study was done on 48.126, 51.873, 56.259 and 64.881 individuals with T2D in p1, p2, p3 and p4 respectively. In p4, the age among the different occupational sectors ranged between 35 and 38 years in the younger age group (18–45 years), and between 55 and 58 years in the older age group (>45 years). Around two thirds of the population were men. Basic population characteristics of the occupational sectors are presented in Table 1.

**Table 1:** Population characteristics.

	2012–2013	2014–2015	2016–2017	2018–2019
N	48126	51873	56259	64881
Age mean (SD)	54 (9)	55 (9)	55 (9)	55 (10)
Gender: % women	33%	33%	34%	35%
Insurance duration days mean (SD)	716 (77)	713 (85)	707 (100)	707 (96)
***Occupational Sector***				
**Agriculture**	959	1085	1183	1331
Gender: % women	15%	16%	18%	18%
Age mean (SD) in 18–45 years	39 (7)	38 (7)	38 (7)	37 (8)
Age mean (SD) in >45 years	56 (6)	56 (6)	56 (6)	57 (6)
**Extraction of raw material, production and manufacturing**	10766	11393	11726	13047
Gender: % women	18%	18%	18%	17%
Age mean (SD) in 18–45 years	39 (6)	38 (6)	38 (6)	38 (6)
Age mean (SD) in >45 years	56 (6)	56 (6)	56 (6)	57 (6)
**Construction, architecture, measuring and building Technology**	3893	4103	4215	4685
Gender: % women	1,4%	1,5%	1,4%	1,4%
Age mean (SD) in 18–45 years	40 (5)	39 (6)	39 (6)	38 (6)
Age mean (SD) in >45 years	56 (6)	56 (6)	56 (6)	57 (6)
**Natural sciences, geography, information**	573	600	649	752
Gender: % women	15%	16%	17%	16%
Age mean (SD) in 18–45 years	39 (6)	38 (6)	38 (6)	37 (6)
Age mean (SD) in >45 years	56 (6)	56 (6)	55 (6)	56 (6)
**Transport, logistics, protection and security**	13528	14729	15908	17919
Gender: % women	31%	30%	30%	30%
Age mean (SD) in 18–45 years	39 (6)	389 (5)	38 (6)	38 (6)
Age mean (SD) in >45 years	57 (6)	57 (6)	57 (6)	58 (6)
**Commercial, trade, distribution and tourism**	2987	3411	3956	4850
Gender: % women	64%	65%	64%	65%
Age mean (SD) in 18–45 years	37 (7)	37 (7)	36 (7)	36 (7)
Age mean (SD) in >45 years	55 (6)	56 (6)	55 (6)	56 (6)
**Corporate organization, accounting, law and administration**	2793	3039	3564	4441
Gender: % women	60%	60%	63%	64%
Age mean (SD) in 18–45 years	36 (7)	36 (7)	37 (6)	37 (6)
Age mean (SD) in >45 years	56 (6)	57 (6)	57 (6)	57 (6)
**Health sector, social work, teaching & education**	4062	4645	5559	6807
Gender: % women	82%	81%	80%	81%
Age mean (SD) in 18–45 years	36 (7)	36 (7)	36 (7)	35 (7)
Age mean (SD) in >45 years	56 (6)	56 (6)	56 (6)	56 (6)
**Humanities, culture and design**	302	334	402	495
Gender: % women	44%	45%	47%	47%
Age mean (SD) in 18–45 years	35 (7)	36 (6)	37 (6)	37 (6)
Age mean (SD) in >45 years	55 (6)	55 (6)	55 (6)	55 (6)

### DCSI-CC in all working men and women

**Fig 1:**
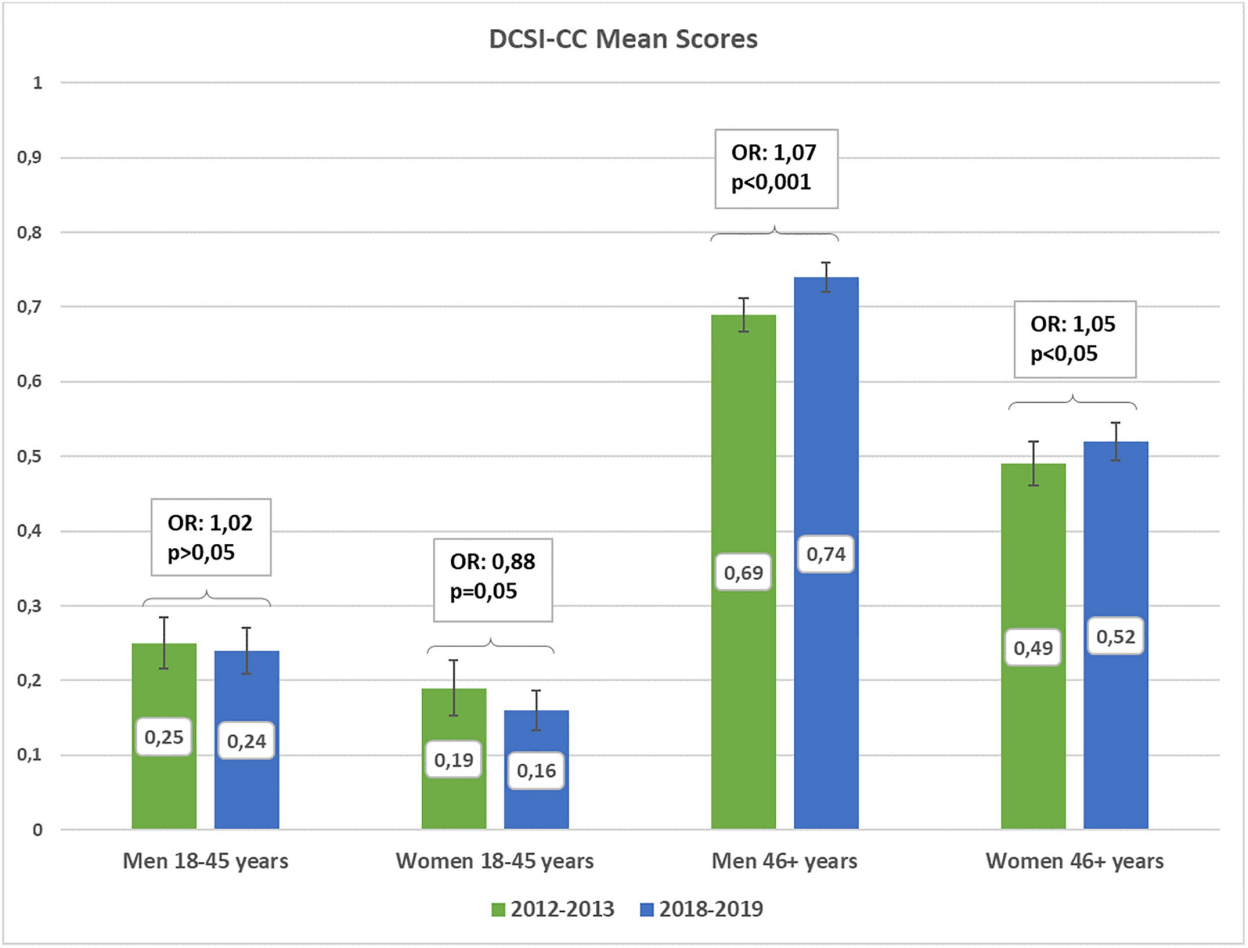
***Bars*:** Means and confidence intervals of DCSI-CC scores in working men and women of the two age groups 18–45 years and 46+ years for the two periods: 2012–2013 (green) and 2018–2019 (blue). ***Boxes*:** P-values for the temporal differences between the two periods (ORs for the odds of having a higher DCSI-CC score in 2018–2019 versus 2012–2013 as the reference category) within each subgroup based on ordinal logistic regression analyses adjusting for age within each age group and duration of observation within each time period, correcting for within cluster variation. Model 1: Men 18–45 years, N = 9849; Model 2: Women 18–45 years, N = 7527; Model 3: Men 46+ years, N = 64745; Model 4: Women 46+ years, N = 30886.

In the working population of individuals with T2D, women of both age groups had lower DCSI-CC mean scores compared to men of the corresponding age groups. In p4, the mean DCSI-CC score for women in the age group 18–45 years was 0,16, compared to 0,24 in men. In the older age group (>45 years), the mean DCSI-CC score was 0,52 in women and 0,74 in men. The temporal development between 2012–13 and 2018–19 exhibited different trends in the two age groups. Though not significant, there appeared to be a potential towards a reduction in the mean DCSI-CC scores in the younger age group. On the other hand, there was a significant increase in the mean DCSI-CC score between p1 and p4 in the older age group in both working men and working women. Men and women of the older age group had a 7% and 5% higher odds of having at least one score point higher in the DCSI-CC score in p4 compared to p1, respectively (Men: OR = 1,07; p<0,001), (Women: OR = 1,05; p<0,05) (Fig 1). The results indicate that older working individuals are having more comorbidities over time.

### DCSI-CC in different occupational sectors

**Fig 2:**
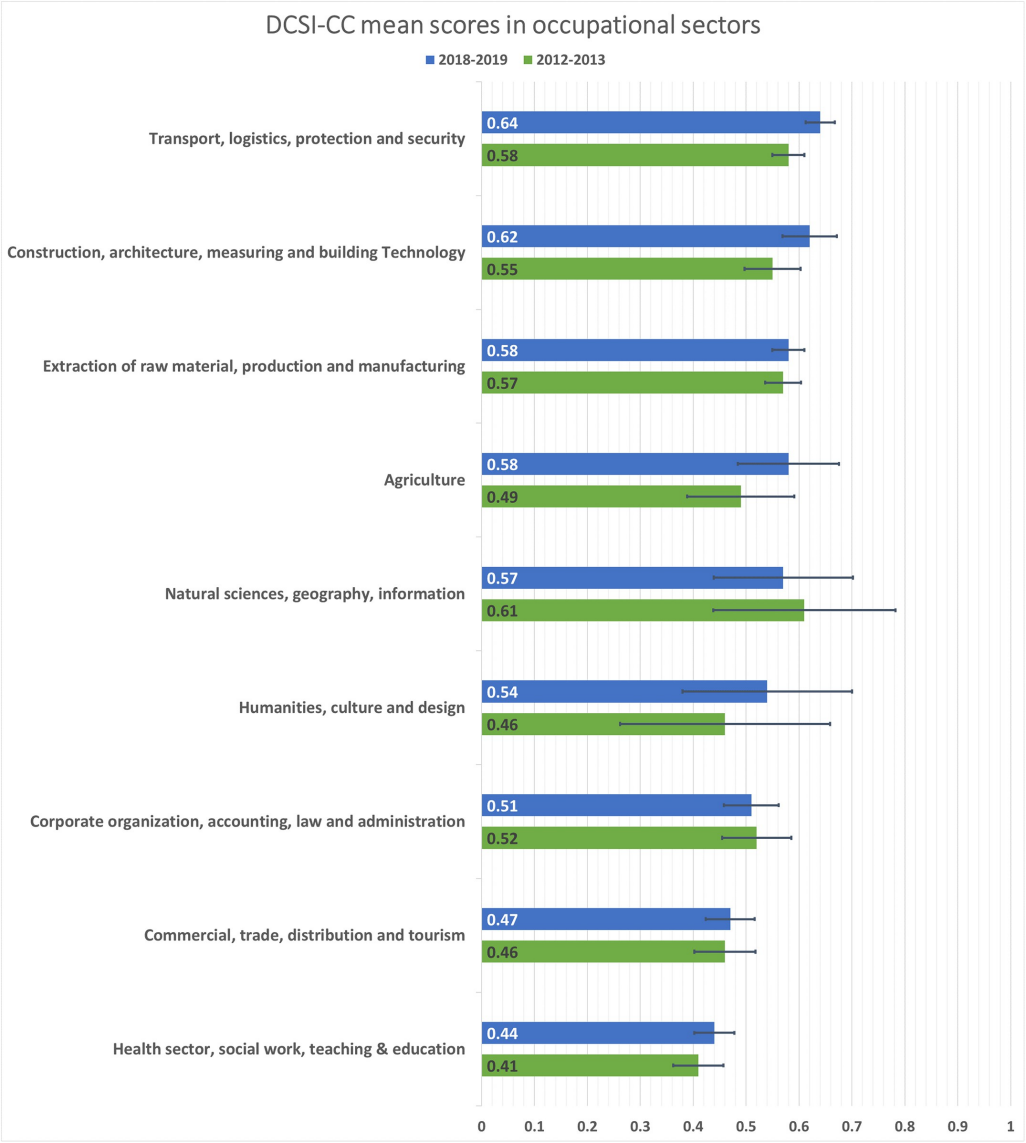
Mean unadjusted DCSI-CC scores and 95% confidence intervals among nine occupational sectors.

The average DCSI-CC score ranged between 0,41 and 0,64 among the different occupational sectors. In p4, men and women working in the occupational sector “Transport, logistics, protection and security” had the highest DCSI-CC average (0,64), followed by the occupational sector “Construction, architecture, measuring and building technology” (mean DCSI-CC = 0,62). On the other hand, the occupational sector “Health sector, social work, teaching and education” and “Commercial, trade, distribution and tourism” had the lowest mean DSCI scores (0,44 and 0,47 respectively) (Fig 2).

### Trends of DCSI-CC in different occupational sectors

Overall, mean DCSI-CC scores were higher in p4 compared to p1 in all occupational sectors except for the two sectors: “Natural sciences, geography, information” and “Corporate organization, accounting, law and distribution”. The sex-stratified analyses revealed that among all sectors, men had higher mean DCSI-CC scores compared to women. Means and ranges of DCSI-CC scores among men and women in different occupational sectors are presented in Table 2.

**Table 2:** Means and ranges of DCSI-CC scores in men and women working in nine occupational sectors and in four time periods between 2012 and 2019.

	2012–2013			2014–2015			2016–2017			2018–2019		
	n	Mean (SD)	Range	n	Mean (SD)	Range	n	Mean (SD)	Range	n	Mean (SD)	Range
**Men**												
Agriculture	811	0,50 (0,79)	0–5	910	0,45 (0,76)	0–5	966	0,51 (0,82)	0–5	1092	0,60 (0,89)	0–5
Extraction of raw material, production and manufacturing	8824	0,59 (0,9)	0–6	9340	0,52 (0,84)	0–6	9641	0,57 (0,87)	0–6	10794	0,60 (0,89)	0–6
Construction, architecture, measuring and building technology	3839	0,55 (0,84)	0–6	4042	0,51 (0,82)	0–6	4157	0,58 (0,86)	0–5	4618	0,63 (0,9)	0–6
Natural sciences, geography, information	486	0,62 (1,05)	0–5	502	0,5 (0,86)	0–5	537	0,59 (0,92)	0–5	633	0,58 (0,9)	0–5
Transport, logistics, protection and security	9400	0,62 (0,92)	0–6	10287	0,56 (0,88)	0–6	11148	0,65 (0,93)	0–6	12641	0,70 (0,96)	0–6
Commercial, trade, distribution and tourism	1087	0,63 (0,94)	0–6	1201	0,56 (0,88)	0–5	1417	0,55 (0,87)	0–5	1718	0,60 (0,89)	0–5
Corporate organization, accounting, law and administration	1117	0,68 (0,99)	0–6	1218	0,63 (0,94)	0–6	1329	0,68 (0,97)	0–5	1594	0,70 (0,99)	0–5
Health sector, social work, teaching & education	745	0,57 (0,88)	0–6	891	0,53 (0,87)	0–5	1091	0,58 (0,88)	0–6	1273	0,64 (0,94)	0–5
Humanities, culture and design	169	0,61 (1)	0–5	184	0,49 (0,8)	0–4	215	0,60 (0,92)	0–4	261	0,69 (1)	0–6
**Women**												
Agriculture	148	0,46 (0,85)	0–5	175	0,43 (0,87)	0–5	217	0,47 (0,88)	0–5	239	0,50 (0,89)	0–5
Extraction of raw material, production and manufacturing	1942	0,45 (0,81)	0–5	2053	0,39 (0,76)	0–5	2085	0,45 (0,79)	0–5	2253	0,46 (0,8)	0–5
Construction, architecture, measuring and building technology	54	0,50 (0,91)	0–4	61	0,51 (0,92)	0–4	58	0,64 (1,02)	0–4	67	0,49 (0,93)	0–4
Natural sciences, geography, information	87	0,54 (1,08)	0–5	98	0,54 (1,03)	0–5	112	0,38 (0,87)	0–5	119	0,50 (0,99)	0–5
Transport, logistics, protection and security	4128	0,48 (0,83)	0–5	4442	0,36 (0,73)	0–6	4760	0,47 (0,81)	0–6	5278	0,52 (0,85)	0–5
Commercial, trade, distribution and tourism	1900	0,36 (0,71)	0–5	2210	0,35 (0,74)	0–5	2539	0,39 (0,76)	0–5	3132	0,4 (0,77)	0–5
Corporate organization, accounting, law and administration	1676	0,41 (0,79)	0–5	1821	0,35 (0,74)	0–5	2235	0,36 (0,74)	0–5	2847	0,40 (0,79)	0–5
Health sector, social work, teaching & education	3317	0,38 (0,74)	0–5	3754	0,33 (0,7)	0–5	4468	0,37 (0,72)	0–5	5534	0,40 (0,74)	0–5
Humanities, culture and design	133	0,26 (0,63)	0–3	150	0,29 (0,68)	0–3	187	0,29 (0,62)	0–3	234	0,37 (0,77)	0–4

**Fig 3:**
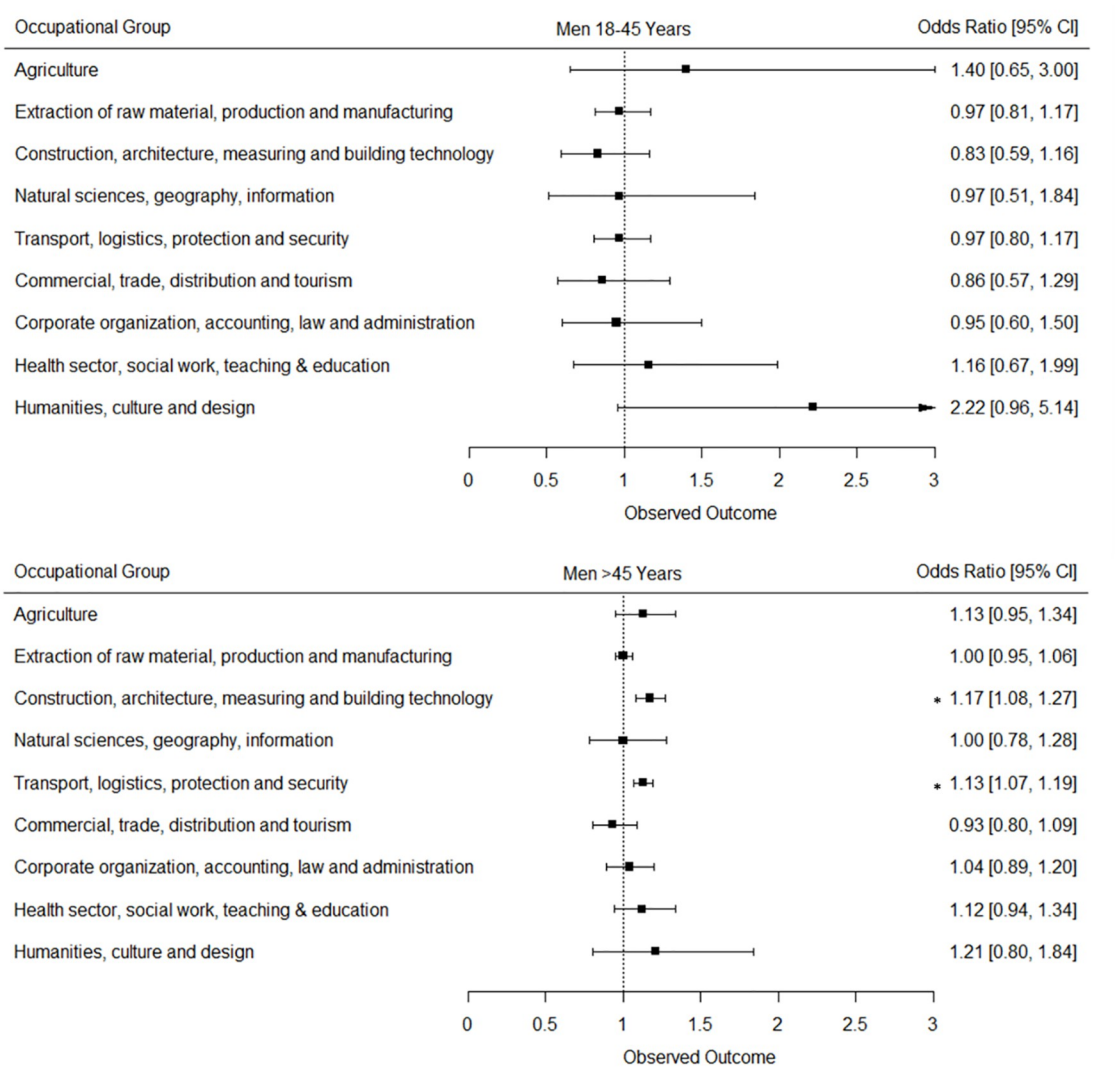
Odds ratios and confidence intervals displaying the odds of having at least one additional DCSI score point in p4 compared to p1 (reference) in men of two age groups working in nine different occupational sectors. Examined by ordinal logistic regression analyses adjusting for age within each age group and insurance duration. p1: 2012–2013; p4: 2018–2019; * p<0,001.

**Fig 4:**
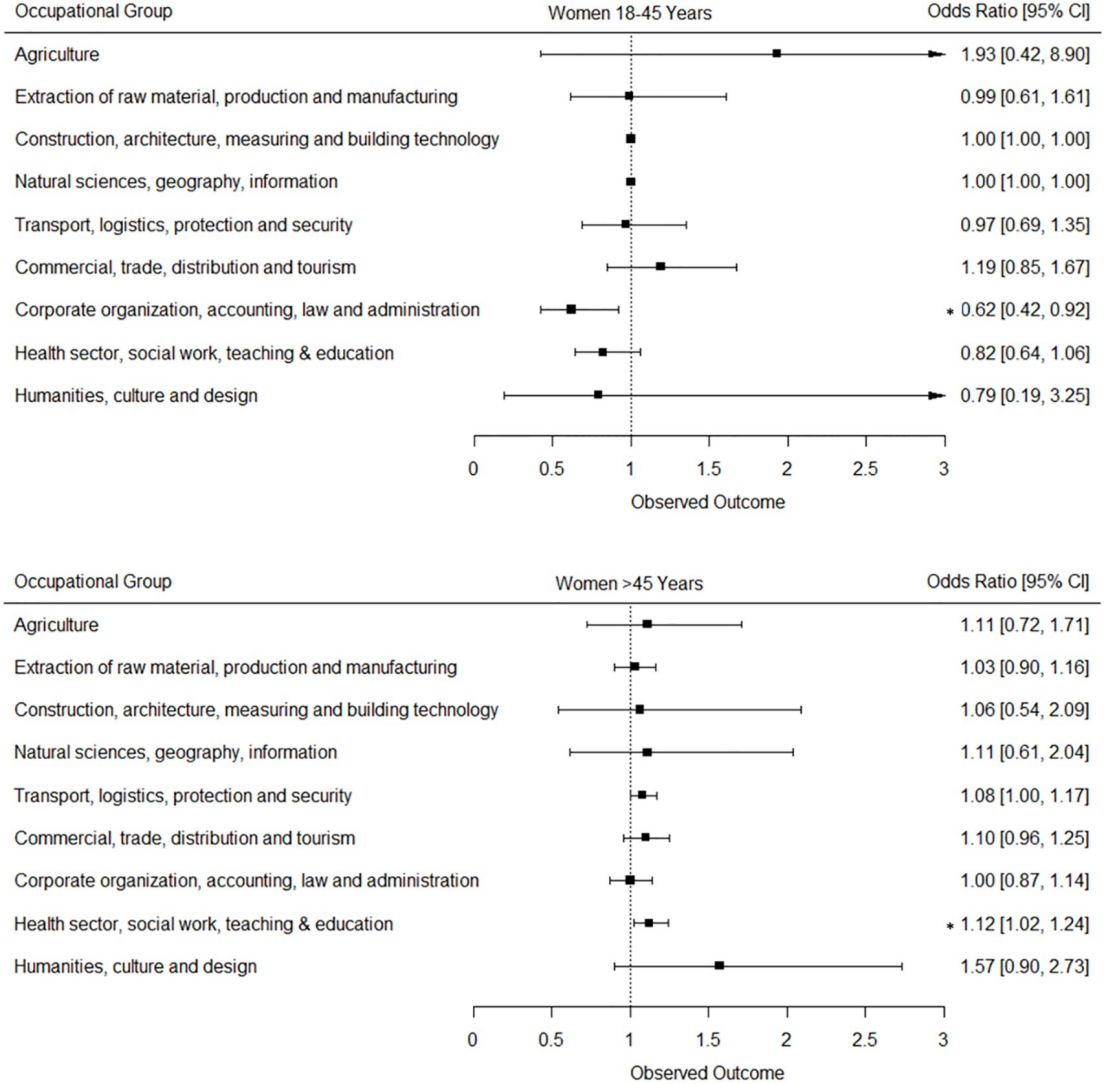
Odds ratios and confidence intervals displaying the odds of having at least one additional DCSI score point in p4 compared to p1 (reference) in women of two age groups working in nine different occupational sectors. Examined by ordinal logistic regression analyses adjusting for age within each age group and insurance duration. p1: 2012–2013; p4: 2018–2019; * p<0,05.

The ordinal logistic regression analyses revealed that among working men and women of the younger age group, even though there was a tendency towards higher DCSI-CC scores, the temporal rise was only significant in younger women working the sector: “Corporate organization, accounting, law and administration”. In the older age group, the temporal rise in DCSI-CC scores was significant only in several occupational sectors. Men working in the occupational sectors “Transport, logistics, protection and security” and “Construction, architecture, measuring and building technology” had 13% (OR = 1,13, p<0,001) and 17% (OR = 1,17, p<0,001) higher odds of having at least one DCSI-CC score point higher in p4 compared to p1 (Fig 3). In women however, even though the DCSI-CC score in the occupational sector “Health sector, social work, teaching and education” was among the lowest, there was a highly significant increase in the odds of having a higher DCSI-CC score in p4 compared to p1 (OR = 1,12, p<0,001) (Fig 4).

## Discussion

The study examined T2D severity and its temporal development in working men and women with T2D and among nine occupational sectors. It revealed that overall, working men had a higher T2D severity compared to working women. In addition, there was a significant rise in T2D severity over time in working men and women of the older age group only. Moreover, the study displayed occupational sector differences in T2D severity. Individuals working in “Health sector, social work, teaching & education” were among the ones to have relatively lower T2D severity, while those working in the sector “Transport, logistics, protection and security” had the highest T2D severity. In these two occupational sectors, T2D severity increased significantly over time.

### Trends in T2D severity in working individuals

In a previous analysis on the working population of the same database with the same time-periods, T2D prevalence appeared to be decreasing over time in older working individuals [15]. Nevertheless, this study revealed that among older working individuals with T2D, T2D severity is increasing over time. These results might indicate that the decrease in T2D prevalence in the population of older working individuals would be due to the increase in the morbidity level, which might lead to exiting the labour market. This is in line with the results of previous research that indicated expansion of morbidity in individuals with T2D [16, 17]. Particularly in employed individuals, the number of comorbidities per individual and the rates of most T2D concordant comorbidities was markedly increasing over time reflecting an expansion of morbidity in this population subgroup [16]. This study added that this specifically applies to the age group of 46+ years and when using another commonly adapted measure of T2D severity, the DCSI-CC. In fact, within the background of demographic ageing, this finding is important as it can have serious implications on the working market and the economic burden of T2D, especially among certain occupational sectors where the increase of T2D severity was pronounced. In line with this argumentation, several studies reported the adverse effects of diabetes on employment and work-related outcomes [36–38]. A previous study done with SHARE data pointed out that in Europe, diabetes as well as cardiovascular diseases are associated with premature losses to the work force due significantly increased probabilities of disability benefits and early retirement in older working individuals [39]. Thus, the rising trend in T2D severity among older working individuals highlights the increased economic burden of this disease and the need to further investigate and manage potential risk factors, also in working environments.

### Differences among occupational sectors

Previous research examining socioeconomic inequalities in the trends of T2D comorbidities done using the same database reported that when considering occupational differences in terms of autonomy and prestige, no differences could be observed in the rates and trends of T2D comorbidities among employed individuals [16]. However, our results indicated that when considering a horizontal stratification of occupation, clear occupational sector differences could be observed in T2D severity as examined by the DCSI-CC, as well as different extents of the temporal development. This emphasizes the importance of considering occupational differences regardless of social class in order to capture and define vulnerable groups.

Among occupational sectors, “Transport, logistics, protection and security” and “Construction, architecture, measuring and building Technology” had the highest T2D severity. Since studies on T2D severity among different occupational groups are limiting, it is challenging to find supporting literature. Nevertheless, the occupational sector “Transport, logistics, protection and security” has been also shown to have the highest rates of prevalent T2D [15], and thus appears to be important vulnerable group. In fact, individuals working in the transport and construction sectors have been shown to have relatively higher risk levels for chronic diseases such as diabetes and cardiovascular disease [40–44]. Moreover, several international studies reported higher metabolic risk profiles as demonstrated by risk factors such as overweight and obesity among workers of these sectors [45–48]. On the other hand, the occupational sector “Construction, architecture, measuring and building technology” had relatively lower rates for T2D as shown in the previous publication [15], but was among the occupational sectors with the highest severity of T2D. This discrepancy could indicate that individuals working in this sector have lower health check-up rates, which would lead to an underestimation of the observed prevalence of T2D. As a result, the higher probability for late diagnosis in this sector could have contributed to the relatively higher T2D severity due to more complications.

Subsequently, our study also indicated that T2D severity is significantly increasing over time in men working in these two occupational sectors, as there was a significant increase in the odds of having at least one additional DCSI-CC score point between 2012 and 2019. Given the reported mean DCSI-CC scores which are under zero (and thus the odds of having at least one whole additional point in the DCSI-CC score is not easy to achieve) and the relatively short observational duration (eight years), this significant temporal development appears to be alarming. Thus, there is a great potential for risk management interventions that need to be realistically personalized to be suitable for the work contexts of individuals working in these sectors. As a prerequisite however, sub-occupations within these sectors need to be closely investigated for work context specific risk factors that need to be addressed.

On the other hand, individuals working in the “Health sector, social work, teaching & education” had lower T2D severity compared to other sectors, even though they were among the mostly affected sectors with respect to T2D prevalence [15]. One key element that could play a role in this specific constellation is health literacy. It could be argued that high stress levels associated with high working hours, sleep deprivation and their effects on physical and mental health in these occupations [49–51] could be associated with relatively higher risks for T2D. At the same time however, individuals working in this sector might have relatively higher educational level and better health literacy [52, 53], which could explain the relatively low severity level in terms of T2D complications due to better knowledge of disease management and glycaemic control. Nevertheless, among women, which also represent around 82% of individuals with T2D working in this sector, T2D severity is significantly increasing over time, making this occupational sector as well a vulnerable one that need to be addressed in further studies.

In men, individuals with T2D working in the occupational sector of agriculture (85% men) had the lowest T2D severity as illustrated by the lowest DCSI-CC scores. This occupational sector also had the lowest T2D prevalence [15]. Despite the fact that individuals working in this sector have relatively a lower educational level [12], which is a strong correlate of health literacy, the results indicate the relatively low vulnerability level of individuals working in this occupational sector. However, though not significant, the time trend analysis indicates a potential towards an increase in T2D severity in this sector, which could be the effect of digitalization on physical activity at work, one important protective factor associated with working in this sector. This needs to be investigated in further studies using longer and more recent observational periods.

Overall, there appeared to be clear gender differences in occupational groups with respect to T2D severity and its temporal development, illustrating a higher risk level for men compared to women. Analyses also revealed a gender specific significant increase in T2D severity over time in different occupational sectors. In fact, numerous studies reported gender differences in the rates of T2D [54, 55], developed T2D complications [17, 56] as well as the quality of T2D care received [57]. This study added that gender differences in T2D severity and its temporal development are evident also in the occupational context. Thus, it would be beneficial to consider gender specific T2D prevention and management in occupational interventions.

### Limitations and strengths

To the best of our knowledge, this is the first study to examine occupational differences in T2D severity. Therefore, we considered broad occupational sectors to provide an overview and evidence on which occupational sectors should be focused upon. examining a narrower classification of occupations within the sectors was beyond the scope of the study due to the several level stratification involved. Results of this study cannot infer causality due to the cross-sectional observation within each time period, and the mixed occupational exposures that could shape metabolic risk profiles within the examined occupational sectors. Further research should consider specific occupations within the vulnerable occupational sectors to identify specific vulnerable groups. Second, potential mediating factors and socioeconomic status were not taken into consideration in this study, since the aim was to describe occupational sector differences regardless of the explanatory mechanisms behind it. Examining potential confounders and mediators requires a second level of analyses that was beyond the scope of this paper. Thus, future studies focusing on specific occupational sectors should consider potential confounders and socioeconomic variations. Moreover, due to the detailed subgroup analysis entailing occupational sector, gender and age group, an index variable to examine T2D severity was used to illustrate clear results. Even though this measure has been shown to be valid in determining T2D severity in several studies, a detailed examination of specific complications would provide a clearer picture. In addition, it cannot be ruled out that diagnoses-coding behaviours in insurance data could have changed over time affecting the trend analysis. However, no clear evidence exists so far on the change in coding behaviour in the examined period. Nonetheless, this study is done on a large population of statutory insured working individuals using claims data that include all diagnoses, limiting potential selection bias associated with the survey design.

## Conclusion

This study provided evidence for the first time on occupational sector differences in T2D severity in Germany. Results of the study imply that certain occupational sectors such as “Transport, logistics, protection and security” and “Construction, architecture, measuring and building technology” are at a higher risk for a more severe T2D as illustrated by higher DCSI-CC scores. A closer look at the affected occupational sectors to examine more specific occupations within is essential to identify vulnerable groups. The population of working individuals with T2D might benefit from occupation and gender personalized T2D management interventions. Overall, the significantly rising trend in T2D severity among older working individuals is alarming and can have serious implications on the working market and the public health burden.

## Supporting information

S1 Table List of ICD-10-GM diagnostic codes for the seven complication groups. (DOCX)

## Abbreviations

AOKN: Allgemeine Ortskrankasse Niedersachsen
DCSI: Diabetes Complications Severity Index
DCSI-CC: Diabetes Complications Severity Index—Complication category Count
ICD-10-GM: German modified 10^th^ version of the International Classification of Diseases
ISCO: International Standard Classification of Occupations
KldB2010: 2010 Classification of Occupations of the German Federal Employment Office
p1; p2; p3; p4: 2012–2013; 2014–2015; 2016–2017; 2018–2019
T2D: Type 2 Diabetes
