# Supplementary material for: Type 2 diabetes severity in the workforce: An occupational sector analysis using German claims data
Source: PLoS One. 2024 Sep 27;19(9):e0309725. doi: 10.1371/journal.pone.0309725 (PMC11432947; doi:10.1371/journal.pone.0309725)
Supplement: S1 Table — (DOCX) [file pone.0309725.s001.docx]

**Manuscript: Type 2 Diabetes Severity in the Workforce: An Occupational Sector Analysis using German Claims Data**

**Table S1.** List of ICD-10-GM diagnostic codes for the seven complication groups

| **Complication group** | **ICD-10-GM code(s)** |
| --- | --- |
| 1. **Ocular** |  |
| Diabetes mellitus with ophthalmic complications | E10.3, E11.3, E12.3, E13.3, E14.3; H28.0, H36.0 |
| Background retinopathy and retinal vascular changes | H35.0 |
| Other specified retinal disorders | H35.8 |
| Degeneration of macula and posterior pole | H35.3 |
| Retinal hemorrhage | H35.6 |
| Other proliferative retinopathy | H35.2 |
| Retinal detachments and breaks | H33.0 |
| Blindness, both eyes and blindness one eye | H54.0; H54.4 |
| Vitreous hemorrhage | H43.1 |
| 1. **Renal** |  |
| Diabetes mellitus with kidney complications | E10.2, E11.2, E12.2, E13.2, E14.2 |
| Glomerular disorders in diseases classified elsewhere | N08.3 |
| Nephrotic syndrome | N04 |
| Chronic nephritic syndrome | N03 |
| Unspecified nephritic syndrome | N05 |
| Chronic kidney disease | N18 |
| Acute kidney failure | N17 |
| Unspecified kidney failure | N19 |
| Mechanical complication of vascular dialysis catheter | T82.4 |
| Renal dialysis | Z49 |
| Dependence on renal dialysis | Z99.2 |
| 1. **Neurological** |  |
| Diabetes mellitus with neurological complications | E10.4, E11.4, E12.4, E13.4, E14.4 |
| Hereditary and idiopathic neuropathy, unspecified | G60.9 |
| Myasthenic syndromes in other diseases classified elsewhere | G73.0 |
| Oculomotor nerve palsy; trochlear nerve palsy; abducent nerve | H49.0, H49.1, H49.2 |
| Mononeuropathy in diseases classified elsewhere | G59.0 |
| Neuropathic arthropathy | M14.6, M14.2 |
| Polyneuropathy in diseases classified elsewhere | G63.2 |
| Autonomic neuropathy in diseases classified elsewhere | G99.0 |
| 1. **Cerebrovascular** |  |
| Transient cerebral ischemic attacks and related syndromes | G45 |
| Intracranial hemorrhage; ischemic stroke; sequelae of those | I61, I63; I64, I69 |
| 1. **Cardiovascular** |  |
| Atherosclerosis | I70 |
| Other acute ischemic heart diseases | I24 |
| Angina pectoris | I20 |
| Chronic ischemic heart disease | I25 |
| Myocardial infarction and complications of MI | I21, I23 |
| Ventricular fibrillation and flutter, cardiac arrest | I49.0, I46 |
| Atrial fibrillation and flutter | I48 |
| Subsequent myocardial infarction | I22 |
| Heart failure, hypertensive heart disease with heart failure, hypertensive heart and chronic kidney disease with heart failure | I50, I11.0, I13.0 |
| Aortic aneurysm and dissection | I71 |
| 1. **Peripheral vascular** |  |
| Diabetes mellitus with circulatory complications | E10.5, E11.5, E12.5, E13.5, E14.5 |
| Other disorders of arteries, arterioles and capillaries in diseases classified elsewhere | I79.2 |
| Aneurysm of artery of lower extremity | I72.4 |
| Peripheral vascular disease, unspecified | I73.9 |
| Embolism and thrombosis of arteries of the lower extremities | I74.3 |
| Atherosclerosis of native arteries of extremities with gangrene or ulceration; diabetic peripheral angiopathy with gangrene | R02, I70.24 |
| Gas gangrene | A48.0 |
| Non-pressure chronic ulcer of lower limb, not elsewhere classified | L97 |
| Non-pressure chronic ulcer of skin, not elsewhere classified | L98.4 |
| 1. **Metabolic** |  |
| Diabetes mellitus with ketoacidosis | E10.1, E11.1, E12.1, E13.1, E14.1 |
| Diabetes mellitus with coma | E10.0, E11.0, E12.0, E13.0, E14.0 |
